# Simultaneous Optimization of Nanocrystalline SnO_2_ Thin Film Deposition Using Multiple Linear Regressions

**DOI:** 10.3390/s140202549

**Published:** 2014-02-06

**Authors:** Saeideh Ebrahimiasl, Azmi Zakaria

**Affiliations:** 1 Department of Nanotechnology, Ahar Branch, Islamic Azad University, Ahar 54515, Iran; 2 Department of Physics, Universiti Putra Malaysia, UPM Serdang 43400, Malaysia

**Keywords:** nanocrystalline SnO_2_, thin film, modeling, ANOVA, energy band gap

## Abstract

A nanocrystalline SnO_2_ thin film was synthesized by a chemical bath method. The parameters affecting the energy band gap and surface morphology of the deposited SnO_2_ thin film were optimized using a semi-empirical method. Four parameters, including deposition time, pH, bath temperature and tin chloride (SnCl_2_·2H_2_O) concentration were optimized by a factorial method. The factorial used a Taguchi OA (TOA) design method to estimate certain interactions and obtain the actual responses. Statistical evidences in analysis of variance including high F-value (4,112.2 and 20.27), very low P-value (<0.012 and 0.0478), non-significant lack of fit, the determination coefficient (R^2^ equal to 0.978 and 0.977) and the adequate precision (170.96 and 12.57) validated the suggested model. The optima of the suggested model were verified in the laboratory and results were quite close to the predicted values, indicating that the model successfully simulated the optimum conditions of SnO_2_ thin film synthesis.

## 1. Introduction

Metal oxide semiconductors with wide band gaps have many important applications in the optics, electric and electronic industries. Transparent SnO_2_ thin films have been widely used in the production of transparent electrodes, far-infrared detectors, solar cells and gas sensors [1–4]. Nanocrystalline SnO_2_ thin films have also garnered attention since higher quality synthesis of SnO_2_ thin films was achieved.

A variety of methods, such as magnetron sputtering [5], vacuum evaporation [6], sol-gel [7], chemical vapor deposition [8], and sonochemistry [9] have been employed to prepare SnO_2_ thin films. Among all the methods, the chemical bath deposition technique is very attractive because it is easy to control the growth factors, and crystal quality [10].

Since in the CBD method several effective parameters such as concentration, time, temperature and the pH of the solution exist, too many experiments must be performed for finding the optimum conditions. Besides laborious experimental management, this also requires more chemicals, instruments and labor time. Furthermore, the preparation conditions are critical factors that affect the shape and size of the resulting nanomaterials [11,12], therefore prediction of the optimal conditions seems highly desirable and necessary.

The Taguchi method is a statistical technique used in empirical studies. It is an conomical way to characterize complicated processes, and it requires fewer experiments to optimize reactions. In the methodology, a factorial design was used for experimental design and fitting the performed results with a polynomial equation in the vicinity of the optimum conditions to make a model [13]. The model relates the responses and the variables of the deposition process [14]. Therefore, in this work the optimum conditions were simulated by the TOA and then visualized by 3D plots in vicinity of the reported optimum conditions [15]. The predicted optima of the responses and the variables were the confirmed by the actual responses from laboratory experiments.

## 2. Experimental

### 2.1. Materials

Glass slides of 76 × 25 mm^2^ were used as the substrate. SnCl_2_·2H_2_O (Merck, Darmstadt, Germany, 98.1%), Ethylenediaminetetraacetic acid and triethanolamine (Sigma Aldrich, St Louis, MO, USA) were used as the complexing agents. Ethanol, HCl and H_2_O_2_ used in this study were of analytical reagent grade.

### 2.2. Empirical Methodology

The reactions were performed in 100 mL flasks and specified volumes of deionized water were added as the solvent. Different concentrations of tin chloride were mixed with the complexing agents, while different amounts of H_2_O_2_, HCl and ethanol were subsequently added to adjust the pH. The reaction was performed in a water bath at different temperatures and for different deposition times, as shown in Table 1. The substrates were preheated to 120 °C and then quickly mounted in cold reaction solution. After that, the reaction vessels were moved to water bath which was maintained at 30–50 °C for 30–90 min. The samples were removed from the bath then dried at room temperature. The surface roughness data was recorded using an atomic force microscope (Quesant Q-scope 250, Ambios Technology, Inc., Santa Cruz, CA, USA). The optical transmission data in the wavelength range of 280–800 nm were recorded using a Lambda 2S Ultraviolet/Visible spectrophotometer (Uberlingen, Germany) at room temperature.

### 2.3. Statistical Methods

To find the optimum deposition conditions, the experiments were designed by factorial and TOA as shown in Table 1 (the design is codified). The design with four effective variables (Table 2) was run by the Design-expert version 8.0.7.1 software (Minneapolis, MN, USA).

The total number of performed runs was nine. The designed actual responses were fitted to the quadratic cubic models by orthogonal array TOA. The fitting was based on a second order polynomial model by a multiple regression analysis [13]. The significance and adequacy of the model was determined by the statistical evidence that appear in analysis of variance (ANOVA) output of the TOA method. These included Fisher variation ratio (F-value), lack of fit, coefficient of determination R-squared (R_d_^2^), adjusted R-squared (R_Adj_^2^), and predicted R-squared (R_Pred_^2^) and adequate precision of Predicted Residual Error of Sum of Squares (PRESS). Most of these parameters are clearly defined in experimental design texts. R_Adj_^2^ and the R_Pred_^2^ are the measurement of the amount of variation around the mean and the new explained data, respectively. F-value is a statistically valid measure of how well the factors described the variation in the data about its meaning while P-value represents the degree of significance of each variable [13].

## 3. Results and Discussion

### 3.1. Analysis of the Model

**Figure 1.**
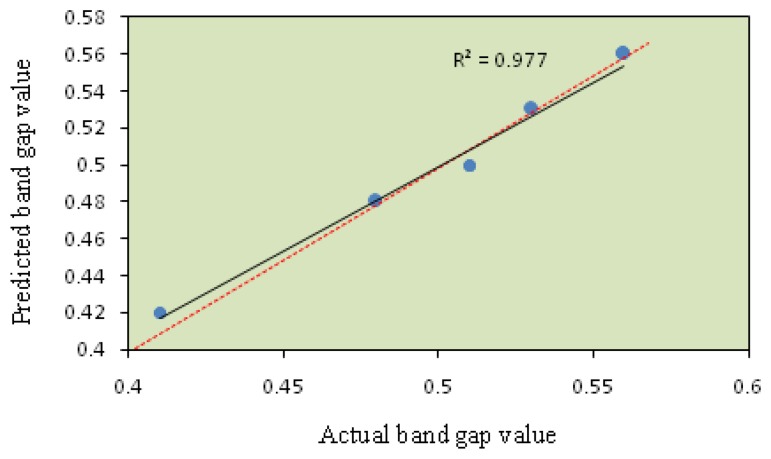
Scatter plot of coded predicted energy band gap value *versus* actual energy band gap value from the Taguchi experimental design.

**Figure 2.**
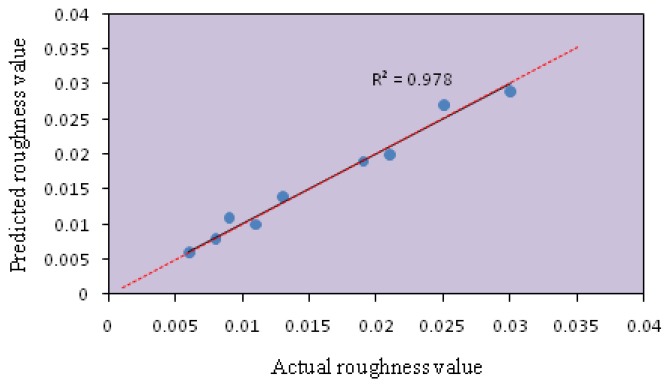
Scatter plot of coded predicted RMS roughness value *versus* actual RMS roughness value from the Taguchi experimental design.

The model fitting technique showed sufficient correlation between the predicted values to the observed values. The fitting of the data to various models (*i.e.*, linear, quadratic, two factorial and cubic) and their subsequent ANOVA showed that a quadratic response surface model was the most suitable to describe the synthesis of nanocrystalline SnO_2_. Figures 1 and 2 show the predicted values *versus* the actual values of the energy band gap and RMS roughness. The coefficients of determination (R^2^) obtained for the energy band gap and RMS were 0.978 and 0.977, respectively.

**Figure 3.**
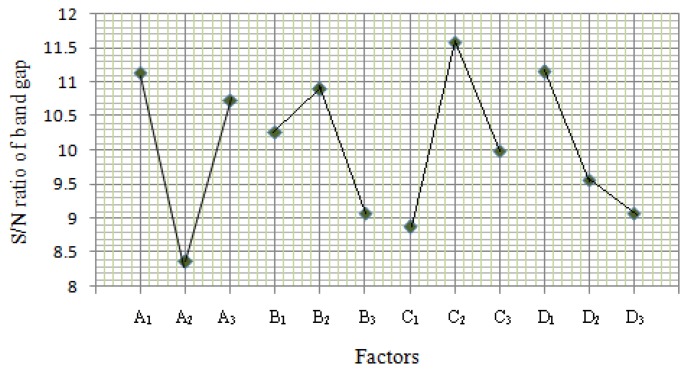
Analysis of effects of control parameters and interaction on S/N for energy band gap.

**Figure 4.**
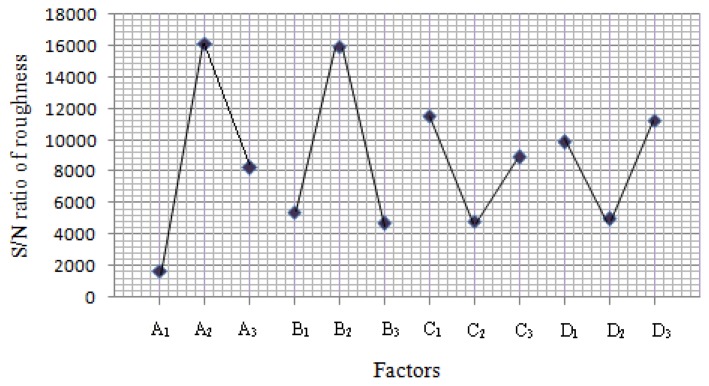
Analysis of effects of control parameters and interaction on S/N for surface roughness.

The S/N ratio is a logarithmic function used to optimize the process or product design and minimize the variability. The maximization of S/N ratio allows reduction of variability of the process against undesirable changes in the neighboring environment or uncontrollable factors. To minimize variability, the level of factor which produces the higher value of S/N ratio must be chosen. The S/N ratios were calculated using Equation (1) [16] as follows:

(1) $$\text{S}/\text{N}=-10\log[\sum_{i=1}^{n}({\text{y}}_{\text{i}}^{2})/\text{n}]$$

where (*y_i_*) denotes the (*n*) observations of response variable. The results of the effects on S/N ratio are shown in Figures 3 and 4. The best conditions of control factors deduced from the signal-to-noise ratio were pH, temperature at level 1, time and concentration at level 2.

The ANOVA results of the quadratic model for the deposition of SnO_2_ thin film are presented in Tables 3 and 4. High F-values of 20.27 and 4,112.2 indicate that the model was significant and there was only a 1.2% and 4.78% chance occurrence of noise.

The coefficients of determination (R^2^) of the model were 0.9838 and 0.9937, which indicated 98.38% and 99.37% of variability in the response could be explained by the model. Therefore, the present R^2^-values reflected a very good fit (>0.9) between the experimental and predicted values [17].

In addition, the R^2^_Adj_ (0.9353 and 0.9997) were satisfactory, which confirms the aptness of the model. Moreover, the adequate precision (12.57 and 170.96) shows remarkable signal (≫4). This ensured model (quadratic) was suitable to navigate the design space and provide a satisfactory match of the polynomial model to the experimental data.

### 3.2. The Quadratic Expression Model

It is normal to describe experimental data by forming a mathematical relationship between the factors (independent variables) and responses (dependent variables). The final model to describe the relationship of the energy band gap and surface roughness with control factors is shown in Equations (2) and (3), respectively, as follows:

(2) $${\text{Y}}_{1}=\;0.44266-\;0.14248\;{\text{X}}_{1}-\;0.00015\;{\text{X}}_{2}+\;0.10042\;{\text{X}}_{3}-0.00346{\;\text{X}}_{4}+\;0.0167\;{{\text{X}}_{1}}^{2}+\;0.00075\;{\text{X}}_{1}\times {\text{X}}_{2}$$

(3) $${\text{Y}}_{2}=2.36793-0.72106\;{\text{X}}_{1}-\;0.00115\;{\text{X}}_{2}+0.46744\;{\text{X}}_{3}-0.00081\;{\text{X}}_{4}+0.063721\;{{\text{X}}_{1}}^{2}+20.81283\;{{\text{X}}_{3}}^{2}+0.79181\;{\text{X}}_{1}\times {\text{X}}_{3}$$

where X_1_, X_2_, X_3_ and X_4_ are demonstrated in Table 2. The linear terms of equations include (X_1_, X_2_, X_3_ and X_4_) shows a direct relationship between the response and the effective factors. Quadratic terms of equation include (X_1_^2^ and X_3_^2^), are one way of obtaining the maximum or minimum value and show the optimum performance at a particular factor. The interaction term (X_1_X_3_, X_1_X_2_) implies the dependent influence of two factors on the response. Negative values of coefficient estimates denote negative influence of parameters on the responses. It was observed that all the linear coefficients of the model gave a negative effect, except the coefficient estimated for concentration (C). This indicates that the energy band gap and surface roughness were negatively affected by pH, temperature and time. Besides, it was also observed that concentration had a significant effect on responses. Therefore the surface morphology and energy band gap of deposited SnO_2_ thin film improve by decreasing the pH, temperature and time to minimum levels, and increasing the concentration to the maximum level of the design.

### 3.3. Response Surface Plots

**Figure 5.**
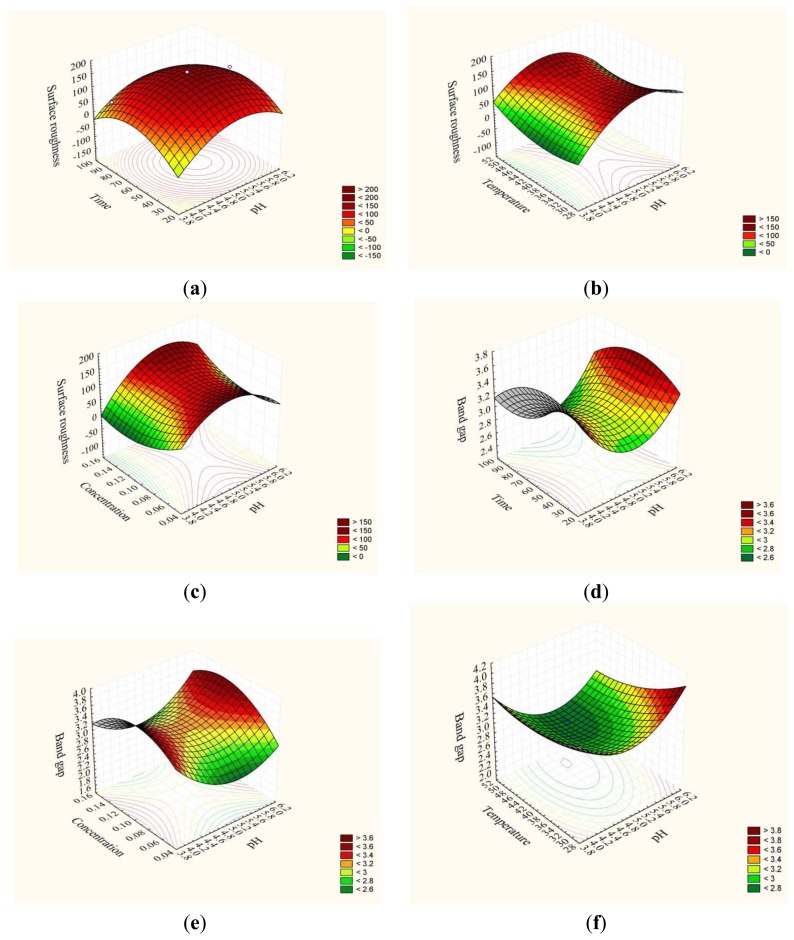
Response surface plots indicating the effect of interaction between process variables on synthesis of nanocrystalline SnO_2_ thin film (**a**) Interaction between time and (**b**) Interaction between pH and concentration (**c**) Interaction between temperature and pH for surface roughness (**d**) Interaction between time and pH and (**e**) Interaction between pH and concentration (**c**) Interaction between temperature and pH for energy band gap.

Based on the validated model, the 3D plots present the numerous predicted (simulated) responses with the four variables and one response (Table 2) of the deposition (Figure 5). The effect of pH, deposition time, temperature and SnCl_2_ concentration on chemical synthesis of SnO_2_ thin film was investigated during the deposition as a preliminary study, while two variables in each case were held constant (e.g., Figure 5a). As observed, the deposition illustrated a peak at a particular value of pH, deposition time, temperature and SnCl_2_ concentration.

Figure 5a shows the result of the mutual interaction of pH and time with constant temperature and concentration (40 °C and 0.1 M). As shown the RMS roughness of the film increases by increasing the deposition time to 55 minute and the pH value to 5.2, since at acidic pH, the concentration of H^+^ ions increases in the solution (Equation (5)) and according to Le Chatelier's principle, this reduces the rate of ionization of H_2_O (Equation (4)) and the crystallization rate accordingly:

(4) $${\text{H}}_{2}\text{O}↔{\text{H}}^{+}+{\text{OH}}^{-}$$

(5) $$\text{HCl}\to {\text{H}}^{+}+{\text{Cl}}^{-}$$

(6) $${\text{SnCl}}_{2}\to {\text{Sn}}^{2+}+{\;2\text{Cl}}^{-}$$

Thus, the nucleation occurs by multiple growths and reduces the size and surface roughness of the film:

(7) $${\text{Sn}}^{2+}+{\text{2OH}}^{-}\to \text{Sn}{\left(\text{OH}\right)}_{2}$$

(8) $$\text{Sn}{\left(\text{OH}\right)}_{2}\to \;\left[{\text{Sn}}^{2+}+\text{2}\left({\text{O}}^{2-}+{\text{H}}^{+}\right)\right]\to \text{SnO}+{\text{H}}_{2}\text{O}$$

(9) $$\text{SnO}+{\text{H}}_{2}{\text{O}}_{2}\to {\text{SnO}}_{2}+{\text{H}}_{2}\text{O}$$

A decrease in RMS roughness was observed above the optimum. The reduction may be due to the prolonged deposition time which destroys the film.

Figure 5b shows the interaction between pH and tin chloride concentration at constant temperature and time (40 °C and 60 min). As shown, the surface roughness decreased slightly as the concentration increased from 0.05 M to 0.1 M in the studied range of pH. The decrease in the surface roughness may be due to increasing Sn^2+^ ions in the reaction solution (Equation (6)) and growth centers on the substrate subsequent (Equation (7)). Further increases in concentration increased the roughness of the film. Excess Sn^2+^ ions would not have the opportunity to react with the OH^−^ ions resulting in cluster growth of the film.

The same trend was observed in Figure 5c for the interaction between pH and temperature at constant concentration and time (0.1 M and 60 min). Generally, as the temperature of the system increases, the number of molecules that carry enough energy to react increases and the rate of reaction increases. Thus the rate of release of precursors and grain growth increase (Equations (4) and (6)), which improves the surface roughness. An optimum temperature point was achieved at 40 °C.

Figure 5d shows the result of the mutual interaction of pH and time with constant temperature and concentration (40 °C and 0.1 M) for the energy band gap. As shown, the energy band gap decreased with increasing pH from pH 4 to 5 over the range of deposition times. This can be related to the deposition of side products, such as SnO and Sn(OH)_2_ (Equations (7) and (8)) which decrease the energy band gap of the deposited SnO_2_ thin film. Further increases again increase the energy band gap value.

Figure 5e shows the result of the mutual interaction of pH and concentration with constant temperature and time (40 °C and 60 min) for the energy band gap. An optimum point for the energy band gap was observed at a concentration of 0.1 M in the range of studied pH values. However, a decrease in energy band gap was observed above and below the optimum.

Figure 5f shows the interaction between pH and temperature with constant concentration and time (0.1 M and 60 min). As observed, an acceptable energy band gap was obtained at lower temperatures. Any increase in the temperature resulted in a diminished energy band gap.

As a result, the optimum conditions for energy band gap and surface roughness are somewhat different. Simultaneous optimization led by the model allowed us to evaluate the optimum conditions for both responses. As a result the optimum point for energy band gap and surface roughness were 3.40 eV (average) and 50 nm under the conditions of pH of 6 and tin chloride concentration 0.14 M with 78 min of deposition time and 45 °C bath temperature. The optimum was validated by performing confirmatory experiments.

### 3.4. Prediction and Confirmation of Properties

**Figure 6.**
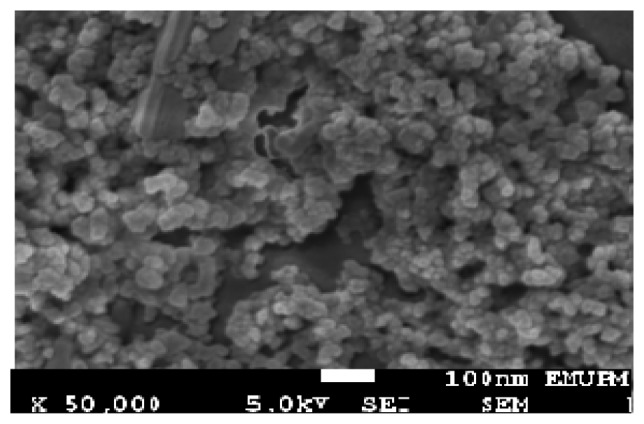
Surface morphology of nanocrystalline tin oxide thin film at the optimum conditions.

**Figure 7.**
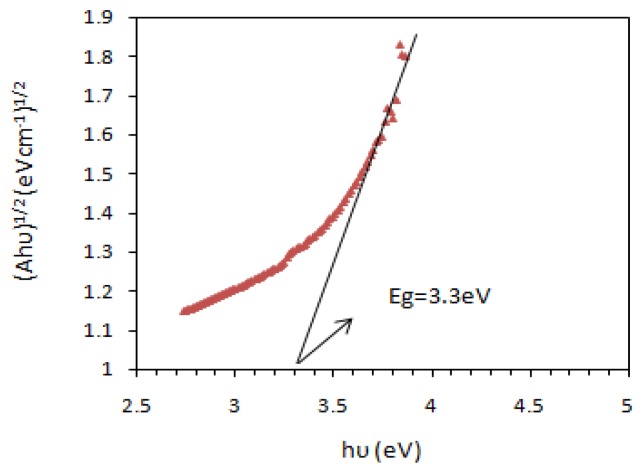
Energy band gap of nanocrystalline tin oxide thin film at the optimum conditions.

Once the optimum level of the design parameters had been selected, the final step was to predict and verify the improvement of the quality characteristic using the optimum level of the design parameters. Figures 6 and 7 show the surface morphology and energy band gap of nanocrystalline SnO_2_ thin film at the optimum conditions predicted by TOA. As observed, the experimental values were reasonably close to the simulated values, which indicated the high validity and adequacy of the model. The grain size of the deposited SnO_2_ film under the optimum conditions according to SEM observation was about 45 nm.

## 4. Conclusions

Nanocrystalline SnO_2_ thin film synthesis was optimized and modeled using a Taguchi robust design method with multiple linear regression analysis. The experiments were designed with four effective factors including concentration, pH value, and deposition time and bath temperature. To suggest a model for the deposition, the responses were fitted with a quadratic model. The ANOVA confirmed the high validity of the model as evidenced of the high F-value (20.27 and 4,112.2), non-significant lack of fit, the R^2^ (98.38% and 99.37%), and the adequate precision (12.57 and 170.96). The results of simulated 3D plots and predicted model for the SnO_2_ nanocrytalline synthesis were in agreement with the experimental results of a confirmation test. This study indicates the success of an orthogonal array to simulate the optimum condition of SnO_2_ nanocrytalline synthesis using the chemical bath method.

## Figures and Tables

**Table 1.** Experimental design of SnO_2_ nanocrystalline thin film deposition

**Std**	**Run**	**X_1_**	**X_2_**	**X_3_**	**X_4_**	**Y** **(BG & SR)** Experimental result
**5**	**1**	1	0	1	−1	-
**9**	**2**	0	−1	0	−1	-
**7**	**3**	0	1	1	0	-
**4**	**4**	1	1	0	1	-
**6**	**5**	1	−1	−1	0	-
**3**	**6**	−1	−1	1	1	-
**8**	**7**	0	0	−1	1	-
**1**	**8**	−1	1	−1	−1	-
**2**	**9**	−1	0	0	0	-

BG: (energy band gap); SR: (surface roughness).

**Table 2.** Independent variables and their levels employed in the factorial design.

	**Variable**	**Units**	**Level of Variables**	

			**Low**	**High**
**X_1_**	pH	-	4	6
**X_2_**	Deposition time	Min	30	90
**X_3_**	Concentration	Mol/L	0.05	0.15
**X_4_**	Temperature	°C	30	50

**Table 3.** Analysis of variance for energy band gap of SnO_2_ nanocrystalline deposition parameters.

**Source**	**Sum of** **Squares**	**Degree of** **Freedom**	**Mean** **Square**	**F Value**	***p* Value**
**model**	0.022268	7	0.003181	4112.205	0.0120
**X_1_**	0.000131	1	0.000131	169.2554	0.0488
**X_2_**	0.002863	1	0.002863	3701.516	0.0105
**X_3_**	0.001045	1	0.001045	1350.768	0.0173
**X_4_**	0.000159	1	0.000159	205.9534	0.0443
**X_1_^2^**	0.008121	1	0.008121	10497.36	0.0062
**X_3_^2^**	0.005415	1	0.005415	6999.376	0.0076
**X_1_ X_3_**	0.001567	1	0.001567	2026.150	0.0141
**Residual**	0.0000007	1	0.0000007	-	-
**Corrected Total**	0.022	8	-	-	-

**R-Squared**		0.9937	**Standard Deviation**		0.00088
**Adjusted R^2^**		0.9997	**Coefficient of** **variation %**		0.18
**Adequate Precision**		170.96	**PRESS**		14

**Table 4.** Analysis of variance for surface roughness of SnO_2_ nanocrystalline deposition parameters.

**Source**	**Sum of** **Squares**	**Degree of** **Freedom**	**Mean** **Square**	**F Value**	***p* Value**
**Model**	0.00055	6	0.00055	20.27	0.0478
**X1**	0.00021	1	0.00021	47.17	0.0205
**X_2_**	0.00005	1	0.00005	10.41	0.0841
**X_3_**	0.00006	1	0.00006	13.26	0.0678
**X_4_**	0.00004	1	0.00004	9.53	0.0909
**X_1_^2^**	0.00022	1	0.00022	49.86	0.0195
**X_1_ X_4_**	0.00006	1	0.00006	12.18	0.0732
**Residual**	0.000009	2	0.000006	-	-
**Corrected Total**	0.0006	8	-	-	-

**R-Squared**		0.9838	**Standard Deviation**		0.0021
**Adjusted R^2^**		0.9353	**Coefficient of** **variation %**		1.34
**Adequate Precision**		12.57	**PRESS**		35
